# Gating and the Need for Sleep: Dissociable Effects of Adenosine A_1_ and A_2A_ Receptors

**DOI:** 10.3389/fnins.2019.00740

**Published:** 2019-07-17

**Authors:** Michael Lazarus, Yo Oishi, Theresa E. Bjorness, Robert W. Greene

**Affiliations:** ^1^International Institute for Integrative Sleep Medicine (WPI-IIIS), University of Tsukuba, Tsukuba, Japan; ^2^Research and Development, VA North Texas Health Care System, Dallas, TX, United States; ^3^Department of Psychiatry, The University of Texas Southwestern Medical Center, Dallas, TX, United States; ^4^Department of Neuroscience, The University of Texas Southwestern Medical Center, Dallas, TX, United States

**Keywords:** adenosine, slow-wave sleep, A_2A_ receptor, A_1_ receptor, slow-wave activity, sleep homeostasis, dopamine, motivation

## Abstract

Roughly one-third of the human lifetime is spent in sleep, yet the reason for sleep remains unclear. Understanding the physiologic function of sleep is crucial toward establishing optimal health. Several proposed concepts address different aspects of sleep physiology, including humoral and circuit-based theories of sleep-wake regulation, the homeostatic two-process model of sleep regulation, the theory of sleep as a state of adaptive inactivity, and observations that arousal state and sleep homeostasis can be dissociated in pathologic disorders. Currently, there is no model that places the regulation of arousal and sleep homeostasis in a unified conceptual framework. Adenosine is well known as a somnogenic substance that affects normal sleep-wake patterns through several mechanisms in various brain locations via A_1_ or A_2A_ receptors (A_1_Rs or A_2A_Rs). Many cells and processes appear to play a role in modulating the extracellular concentration of adenosine at neuronal A_1_R or A_2A_R sites. Emerging evidence suggests that A_1_Rs and A_2A_Rs have different roles in the regulation of sleep. In this review, we propose a model in which A_2A_Rs allow the brain to sleep, i.e., these receptors provide sleep gating, whereas A_1_Rs modulate the function of sleep, i.e., these receptors are essential for the expression and resolution of sleep need. In this model, sleep is considered a brain state established in the absence of arousing inputs.

## Introduction

Sleep is a highly conserved behavior that is vital to survival among all living organisms with a nervous system, from worms to humans. Chronic sleep loss is linked to a wide range of deleterious physiologic changes, such as altered food intake, weight loss or gain, skin lesions, compromised thermoregulation, and even death (Rechtschaffen et al., 1989; Siegel, 2008). Humans spend roughly one-third of their lives asleep. While we know why we eat, drink, and mate, we do not yet know why we sleep. The neuroscience community has therefore increased efforts to gain knowledge of the physiologic function of sleep.

During sleep, cortical neurons alternate between periods of firing and periods of silence. The switching between the two states, also known as ON and OFF states, is widely synchronized across neurons and represented by slow wave activity (SWA) in encephalography. SWA is observed as slow, oscillatory neocortical activity (0.5–4.5 Hz) that intensifies in correlation with wake duration and declines during sleep, but is expressed only during slow wave sleep (SWS). Because SWS-SWA increases as sleep loss is prolonged and decreases as sleep progresses, it is widely used as a marker of mammalian sleep homeostasis. The rates of SWA build-up and decay can be altered by extreme sleep loss or by pharmacologic or genetic manipulations in mammals, especially those affecting adenosine systems of the central nervous system (CNS). The adenosine system can also affect the gating of SWS-SWA expression by modulating the arousal level, thereby altering the duration of time during which sleep homeostasis and function can occur.

Adenosine is the key building block of every cell’s energy source, i.e., adenosine triphosphate (ATP), and the related adenosine mono- and di-phosphates (AMP and ADP, respectively). Adenosine fulfills a wide range of physiologic and pathophysiologic functions (Fredholm, 2014). In the nervous system, adenosine acts as a neuromodulator through metabotropic receptors. Although adenosine acts on four evolutionarily well-conserved receptors present on most cells, it is believed to modulate sleep need and arousal by acting through A_1_ and A_2A_ receptors (A_1_Rs and A_2A_Rs), respectively.

In light of the emerging roles of adenosine and its receptors in regulating different aspects of sleep, we propose a model for the gating and function of sleep. In our model, A_2A_Rs allow the brain to sleep, i.e., these receptors provide sleep gating, whereas A_1_Rs modulate the function of sleep, i.e., these receptors are essential for the expression and resolution of the sleep need.

## Aspects of Sleep/Wake Regulation

### Humoral Theory of Sleep-Wake Regulation

The humoral theory of sleep-wake regulation posits that during wakefulness, one or more endogenous somnogenic factors is produced and accumulated. Brain activity decreases when the concentration of somnogenic substances increases to a certain threshold. These substances are gradually metabolized during sleep, which leads to a return to the waking state. Rosenbaum (1892) hypothesized that sleep is regulated by humoral factors; i.e., excess water accumulation due to oxidative processes in nerve cells during wakefulness depresses neuronal excitability and removal of the excess water during sleep restores full brain activity, resulting in wakefulness. A few years later, Ishimori (1909) and Legendre and Pieron (1913) independently demonstrated the existence of sleep-promoting hypnogenic substances, also known as “hypnotoxins,” in the cerebrospinal fluid of sleep-deprived dogs (Kubota, 1989; Inoué et al., 1995).

The hypnotic effect of adenosine in the mammalian brain was discovered in 1954 (Feldberg and Sherwood, 1954). Adenosine as a neuromodulator with somnogenic properties should thus be classified as a sleep substance. Extensive evidence also suggests that components of the immune system, such as pro-inflammatory cytokines (Krueger et al., 1984, 2001; Mullington et al., 2000, 2001; Krueger and Majde, 2003) [for review, see (Krueger et al., 2011)] and prostaglandins (Ushikubi et al., 1998; Lazarus et al., 2007; Urade and Lazarus, 2013; Oishi et al., 2015) [for review, see (Urade and Lazarus, 2013)], are interrelated with the regulation of sleep. The involvement of other putative hypnogenic substances, including anandamide (Garcia-Garcia et al., 2009), urotensin-II peptide (Huitron-Resendiz et al., 2005), and the *Drosophila* peptide NEMURI (Toda et al., 2019), is also implicated in the sleep process.

### Circuit-Based Theories of Sleep-Wake Regulation

A slow humoral process, however, cannot sufficiently explain the reversibility of sleep, especially rapid transitions from sleep to wake in response to external stimuli. Experimental work by Constantin von Economo in the early 20th century produced findings that inspired circuit-based theories of sleep/wake regulation. In 1916, von Economo began to see patients with a new type of encephalitis eventually referred to as encephalitis lethargica or von Economo’s sleeping sickness. The disorder was characterized by lesions in the anterior hypothalamus leading to prolonged insomnia or lesions at the junction of the brainstem and forebrain leading to prolonged sleepiness (von Economo, 1917; Economo, 1930). Von Economo concluded that these brain areas must play a role in sleep/wake regulation. The “passive theory,” which dominated in the 1940/1950s, suggested that sleep occurs passively due to decreased activity of the brainstem reticular formation (Bremer, 1938; Moruzzi and Magoun, 1949). Importantly, this “passive theory” implicates a necessary active neuronal modulation to maintain a behavioral state of wake via the ascending reticular activating system. Although overly restrictive to the reticular activating system with regard to the wake-modulatory components, the principle of a necessary activation for wake cannot be ruled out; nor can an active sleep-promoting modulation be ruled out, as these are not mutually exclusive types of modulation.

Many decades later, neurons that are active when animals sleep were identified in the ventrolateral preoptic area (VLPO) near the third ventricle in the anterior part of the hypothalamus (Sherin et al., 1996; Chung et al., 2017). Studies demonstrated that sleep is promoted by projections from the GABAergic preoptic area (POA), including the VLPO, to the tuberomammillary nucleus (TMN) in the posterior hypothalamus (Sherin et al., 1998; Chung et al., 2017), which contains neurons that produce histamine, a neurotransmitter having an important role in arousal (Huang et al., 2006; Haas et al., 2008; Oishi et al., 2008). These findings provided strong evidence of sleep control by the POA-TMN neural pathway.

Neural circuits in the brainstem and basal ganglia also regulate sleep/wake behavior. The parafacial zone (PZ) in the medulla contains sleep-promoting GABAergic neurons (Anaclet et al., 2012, 2014) that project to the parabrachial nucleus (PB), a critical nucleus for cortical activation as lesions of the PB result in a comatose state (Fuller et al., 2011).

More recently, the involvement of dopaminergic neurons in the ventral tegmental area (VTA) was strongly implicated in the arousal effect (Eban-Rothschild et al., 2016; Oishi et al., 2017a). A role for dopamine in arousal is also supported by evidence that amphetamine, which induces the release of monoamines (including dopamine), increases alertness and psychomotor performance in sleep-deprived individuals (Bonnet et al., 2005). Dopamine transporters are necessary for the wake-promoting effects of amphetamine (Wisor et al., 2001). Ablating or suppressing GABAergic neurons in the ventral medial midbrain/pons (VMP), including the VTA, produces wakefulness and prevents sleep mainly through dopaminergic systems (Takata et al., 2018; Yu et al., 2019). Furthermore, the ability of dopamine neurons in the VTA to promote wakefulness is at least in part mediated by projections to the nucleus accumbens (NAc; Eban-Rothschild et al., 2016). Medium spiny GABAergic neurons in the NAc can be divided into two groups that respond differentially to stimulation by dopamine or adenosine. Direct pathway neurons express excitatory dopamine D_1_ receptors and inhibitory adenosine A_1_Rs, whereas neurons of the indirect pathway express inhibitory dopamine D_2_ receptors and excitatory A_2A_Rs. In fact, recent studies showed that NAc direct pathway neurons induce wakefulness (Luo et al., 2018) and A_2A_R-expressing indirect pathway neurons strongly induce SWS (Oishi et al., 2017b). The indirect pathway neurons in the NAc produce sleep by inhibiting the ventral pallidum (VP) in the basal forebrain (BF), although these neurons also have sparse to moderate projections to other well-known arousal-promoting areas, such as the lateral hypothalamus, which produces orexin, the TMN, and the VTA. Interestingly, chemogenetic activation of the BF, including the VP, largely reduces sleep (Anaclet et al., 2015).

Altogether, it is impossible to abolish sleep completely by lesioning the afore-mentioned inhibitory circuits, including the POA-TMN, PZ-PB, and NAc-VP pathways, making it unlikely that the regulation of sleep time depends on a single center (i.e., a master switch for sleep in the brain may not exist). On the contrary, the existence of various neural circuits controlling sleep suggests that sleep is gated by different processes. All of these sleep/wake circuits clearly modulate an animal’s level of arousal (i.e., vigilance) to determine the behavioral state and GABA is the key neurotransmitter for promoting the transition from wake to sleep and the duration of sleep. For example, the observation that the level of wakefulness is regulated by VMP GABAergic neurons (Takata et al., 2018) indicates the ability of the brain to adapt an animal’s sleep/wake time to its behavior.

The transition from waking to sleep may be essential, at least under physiologic conditions, for the facilitation of sleep function to occur. A sufficiently increased level of arousal, as may be experimentally or environmentally induced, prevents sleep occurrence and, accordingly, sleep function. The resulting increase in sleep need is normally reflected by an increase in SWA in the ensuing sleep episode. During this ensuing episode, SWS-SWA resolves toward a non-sleep deprived baseline and the threshold for arousal to waking decreases. As a matter of fact, local sleep, i.e., a phenomenon in which discrete regions of cortical neurons go “offline” similar to during sleep, but other regions do not, is insufficient for sleep function to occur (Vyazovskiy et al., 2011), most likely as a result of the brain’s massive interconnectivity. Thus the integration of local sleep events into a global sleep state is necessary for effective sleep function, even at a local level.

### Homeostatic Regulation of Sleep (Two-Process Model)

In 1982, Alexander Borbély at the University of Zürich in Switzerland proposed a two-process model of sleep regulation (Borbély, 1982) that currently prevails as a major conceptual framework in sleep research. In a simplified version of the two-process model of sleep regulation, sleep propensities in homeostatic and circadian processes are commonly plotted against the time of day and interactions of the two processes determine the cardinal aspects of sleep regulation. The “homeostatic” process is controlled by the sleep pressure or need that builds up during the waking period and dissipates during sleep. In contrast, the “circadian” process, i.e., the sleep/wake cycle during the day and night, is controlled by a circadian pacemaker or biologic clock. Although it was originally hypothesized that the circadian process is independent of prior sleep and waking, experiments in mice lacking clock genes revealed that clock gene knockout (KO) disrupts not only circadian processes, but also sleep homeostatic processes (Franken, 2013). This suggests overlapping functions for the circadian genes in sleep homeostatic control.

Consistent with the two-process model, a homeostatic response to sleep loss, namely sleep rebound in an animal after sleep deprivation, is considered an essential criterion of sleep. Rebound can reflect an increase in SWS-SWA power and/or an increase in SWS duration along with an increase in consolidation. Of these two rebound parameters, SWS-SWA is better correlated with prior waking time. Although a rebound increase in SWA during SWS is often associated with an increase in SWS time or consolidation, its occurrence may be dissociated from an effect on sleep time (Douglas et al., 2007; Bjorness et al., 2009; Suzuki et al., 2013). Importantly, SWA during SWS is considered to be an indicator of sleep intensity (Borbély and Neuhaus, 1979), providing a dimension beyond time in the recovery from prolonged waking. Interestingly, although sleep rebound is widely observed after sleep loss, some species skip sleep in favor of migration, mating, or other social interactions and do not catch up on lost sleep (Berger and Phillips, 1994; Rattenborg et al., 2004; Lyamin et al., 2005; Fuchs et al., 2009; Thimgan et al., 2010; Lesku et al., 2012). Recently, scientists at the Imperial College London demonstrated that male flies in the presence of another male fly undergo sleep loss that results in a sleep rebound once the male intruder is removed, whereas a resident fly also loses sleep in the presence of a female fly, but shows no sleep rebound when the female fly is removed (Beckwith et al., 2017), suggesting that sexual arousal in flies prevents a homeostatic response to sleep loss. Altogether, there is ample evidence in nature challenging the view that sleep rebound is an inescapable outcome of sleep loss. Nevertheless, the “rebound” in these cases refers only to sleep duration and not to SWS-SWA intensity. This potential dissociation of SWS duration from SWS-SWA expression (i.e., SWS duration may not reflect the SWA changes shaping sleep homeostasis) suggests that it may not be possible to fully interpret rebound sleep or the lack thereof in the absence of SWS-SWA assessment.

Another limitation of the two-process model is that it defines circadian input as the only allostatic component that drives the balance between waking and sleep. Sleep/wake behavior is also influenced by cognitive and emotional factors (Saper et al., 2005; Fernandez-Mendoza et al., 2014; Mullins et al., 2014) or other basic drives, such as a lack of food, predator confrontation, mating pressure, and seasonal migration (Yamanaka et al., 2003; Cano et al., 2008). The mechanisms by which motivational stimuli or stressors interact with sleep/wake behavior are not easily accounted for by the two-process model. On the other hand, if the exceptional conditions mentioned above primarily affect arousal level and thus gating of sleep, then sleep homeostasis, conceptualized as “process S” in the two-process model, may still occur. Sleep homeostasis, although related to arousal (sleep need can dissipate to the largest extent only during sleep) appears to follow an exponential rate of decay (Franken et al., 2001; Bjorness et al., 2016). With greater sleep need, there is greater rebound SWS-SWA, but the rate of decay is slowed, further enhancing the amount of SWS-SWA expressed (Bjorness et al., 2016).

The increased sleep duration and consolidation associated with increased sleep need may reflect a decreased level of arousal needed for waking although both external sensory input as well as the internal state (likely to include circadian drive, need for food, predator threat, sex drive, etc.) remain as effective determinants. Accordingly, level of arousal and sleep duration are dynamic, relying on the integration of multiple factors in addition to previous waking time.

### Sleep as a State of Adaptive Inactivity

An alternative view proposes that sleep enforces adaptive inactivity to conserve energy when activity is not beneficial (Siegel, 2009). The wide variability in sleep duration across the animal kingdom (Preston et al., 2009) suggests the sleep amount of an animal may be adapted to the species’ behavior that is critical for survival. Consequently, animals may have the ability to dispense with sleep when varying ecologic demands favor wakefulness; e.g., the ability of male pectoral sandpipers to maintain high neurobehavioral performance despite greatly reduced sleeping time when competing for mating opportunities in a short annual window of female fertility (Lesku et al., 2012) may contradict the notion that decreased performance is an inescapable outcome of sleep loss. A model of sleep as a state of adaptive inactivity challenges the hypothesis that the sleep state persists because it has a vital physiologic function and proposes that sleep has not evolved for what happens when we are asleep, but rather for the energy-saving absence of activity during sleep. The magnitude of energy savings gained through sleep is still unknown, although a new framework for determining relative energy savings during sleep was recently described (Schmidt et al., 2017).

The teleological problem of sleep function may arise from the presumption of sleep’s evolution from a default state of waking. Humans are likely biased toward this presumption by the egocentricity of waking consciousness. The adaptive inactivity model could be modified by reorientation of the question “why do we sleep” to “why do we wake?” In this model, sleep is considered the state that facilitates vegetative functions like anabolism and replacement of proteins, complex carbohydrates, and complex lipids and organelles. The vegetative functions are clearly not inactivity or passive in terms of energy conservation. On the contrary, there is evidence for increased energy utilization in sleep, such as ATP mobilization and AMP dephosphorylation (Dworak et al., 2010). Moreover, the cellular metabolism of brain tissue does not coincide with the systemic eating and digestion of food. From this perspective, an organism is driven to waking primarily by non-vegetative, life-essential pursuits, such as foraging for food, avoiding predators, and, occasionally, sex, along with an integrated circadian timer (also controlling arousal). This model is thus consistent with an active drive or activating system needed to maintain wake.

### Dissociation of the Arousal State and Sleep Homeostasis

An increase in the response threshold to external stimuli is a core feature of sleep (Zepelin, 1994) and is critical for defining sleep in animals that lack a cortex (for review, see Ho and Sehgal, 2005). Prolonged waking by sleep deprivation increases the arousal threshold during subsequent sleep (Williams et al., 1964; Bonnet, 1985), and stronger stimuli are necessary to prevent sleeping/prolong waking (Blumberg et al., 2004). As with spontaneous waking, arousal during sleep deprivation is modulated by internal and external stimuli, but within the context of sleep deprivation the arousal threshold is typically increased. Arousal-related brain regions show greater activity, as measured by c-fos, under sleep deprivation mediated by exposure to novel environments or social interaction compared with gentle handling alone (Deurveilher et al., 2013). Furthermore, the nature of the homeostatic response to prolonged waking varies across development with increases in sleep time preceding the appearance of increased SWA power (Frank et al., 1998). Finally, SWA power, commonly used as an indicator of homeostatic sleep need, is increased within waking during prolonged sleep deprivation (Franken et al., 2001; Vyazovskiy et al., 2011), but the relationship between SWA and sleep homeostasis can be dissociated in pathologic disorders. SWA power is increased during waking and rapid eye movement (REM) sleep in Alzheimer’s disease (Hassainia et al., 1997; Jeong, 2004); whereas SWA is increased during waking but decreased during sleep in schizophrenia (Keshavan et al., 1998; Hoffmann et al., 2000; Fehr et al., 2003). Conversely, faster EEG activity, such as beta and gamma rhythms commonly used as an indicator of arousal, is high during sleep in a subset of insomniacs (Perlis et al., 2001; Dolsen et al., 2017).

## Adenosine and Sleep

### Adenosine Metabolism and Levels During Sleep and Wakefulness

Hydrolysis of AMP and S-adenosylhomocysteine (SAH) produces adenosine (Schrader, 1983; Fredholm, 2007). Adenosine is generated from SAH by SAH hydrolase, which also acts to trap adenosine in the presence of excess L-homocysteine. This takes place intracellularly and the bidirectional actions of the enzyme ensure the constant presence of a particular concentration of adenosine in the cell. Whether SAH hydrolase is involved in generating adenosine in the brain, however, is controversial (Latini and Pedata, 2001). Adenosine is formed intracellularly and extracellularly from 5′-AMP by different 5′-nucleotidase (5′-NT) (Zimmermann, 2000). A cascade of actions by an ecto-5′-NT, together with ecto-ATPases, terminates the action of ATP as extracellular signaling molecules (Zimmermann, 2000, 2006; Yegutkin, 2008; Kovacs et al., 2013).

**FIGURE 1:**
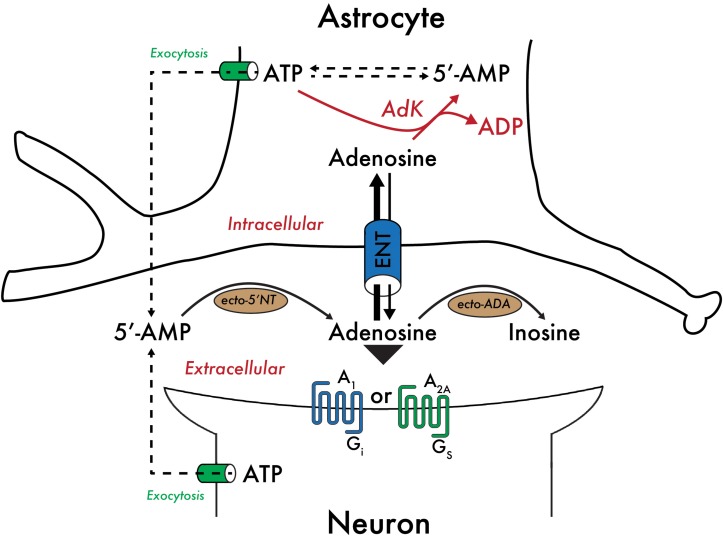
Control of the adenosine concentration by the metabolic state of astrocytes. Adenosine taken up by astrocytes is rapidly phosphorylated to AMP by adenosine kinase (AdK), an enzyme expressed predominantly in glia in the adult CNS. AdK effectively controls the intracellular adenosine concentration by catalyzing the transfer of a phosphate group from ATP to adenosine to produce ADP and AMP. As a result, the rate of adenosine metabolism is reflected by the [ATP]/[ADP][AMP] ratio, linking the rate of adenosine metabolism to the metabolic state of the cell. Equilibrative nucleoside transporters (ENT) bi-directionally regulate the concentration of adenosine available to pre- and post-synaptic A_1_Rs and A_2A_Rs. Other abbreviations used: 5′-NT, 5′-nucleotidase; ADA, adenosine deaminase.

High adenosine levels are reduced by the actions of adenosine deaminase (ADA), or are taken up by cells where adenosine is rapidly phosphorylated to AMP by adenosine kinase (AdK), an enzyme that effectively controls the intracellular adenosine concentration (Figure 1; Fredholm et al., 2005; Oishi et al., 2008; Parkinson et al., 2011). Importantly, AdK binds a molecule of ATP and adenosine, catalyzes the transfer of a phosphate group from ATP to adenosine and produces ADP and AMP. As a result, the rate of adenosine metabolism is reflected by the [ATP]/[ADP][AMP] ratio, linking the rate of adenosine metabolism to the metabolic state of the cell. In the adult CNS, AdK expression occurs predominately in the glia (Studer et al., 2006), and thus the concentration of adenosine is controlled by the metabolic state of the glia.

Bi-directional equilibrative nucleoside transporters regulate the concentration of adenosine available to cell surface adenosine receptors (Parkinson et al., 2011; Dos Santos-Rodrigues et al., 2014). Therefore, adenosine levels are dependent on the formation and removal of extracellular adenosine. Extracellular adenosine levels are low under basal conditions – approximately 30 to 300 nM (Ballarin et al., 1991), but may exceed 1 μM under more extreme conditions, such as mild hypoxia or strenuous exercise, and can reach up to several tens of micromolar concentration in severely traumatic situations, including local ischemia (Fredholm, 2007).

Adenosine triphosphate depletion and an increase of extracellular adenosine levels are positively correlated (Kalinchuk et al., 2003) and positively associated with sleep (Rainnie et al., 1994; Porkka-Heiskanen et al., 1997). Thus, adenosine may represent a state of relative energy deficiency. During spontaneous sleep/wake behavior in cats, adenosine levels in several brain regions are higher during SWS than wakefulness (Porkka-Heiskanen et al., 1997, 2000). Moreover, *in vivo* microdialysis studies in cats revealed that adenosine concentrations increase 2-fold in the BF during a prolonged 6-h period of wakefulness compared with that at the beginning of sleep deprivation. Under more chronic sleep deprivation protocols, however, increases in adenosine concentrations during prolonged waking are no longer observed (Clasadonte et al., 2014), suggesting that loss of the adenosine response may mark a shift from a homeostatic response to an allostatic response following reduced sleep.

Six decades after the discovery of adenosine’s role in sleep, the mammalian brain cell types involved in the sleep-promoting effects of adenosine remain unclear (Feldberg and Sherwood, 1954). ATP, which is rapidly degraded to adenosine, and adenosine are released from glial cells and neurons. In genetically engineered mice in which the release of ATP is non-specifically blocked in astrocytes by selective expression of a dominant negative SNARE domain, decreased concentrations of extracellular adenosine are observed (Pascual et al., 2005). While the amounts of wakefulness, SWS, and REM sleep in these mice are indistinguishable from those in wild-type mice, these mice exhibit reduced SWA and recovery sleep after sleep deprivation (Halassa et al., 2009). Furthermore, reducing AdK in astrocytes, thereby increasing the adenosine tone, is sufficient to increase SWS-SWA and sleep consolidation, reduce the decrease in SWA across the light phase, and slow the decay of SWS-SWA within an average SWS episode, whereas selectively reducing AdK in neurons has no effect (Bjorness et al., 2016). These observations suggest that adenosine mediates the sleep deprivation-induced homeostatic sleep response. The source of the released adenosine, however, remains controversial. Some of the adenosine may originate from astrocytes and the majority may originate from neurons, but direct proof is lacking and thus the exact source of adenosine remains unknown. On the other hand, control of extracellular adenosine modulating sleep need clearly involves glial metabolism mediated by AdK (Bjorness et al., 2016).

Radulovacki et al. (1983) extensively investigated the effects of adenosine on wakefulness. They found that increasing the levels of adenosine in the central nervous system of rats by systemic administration of the ADA inhibitor deoxycoformycin led to increases in REM and SWS. In addition, Oishi et al. (2008) reported that focal administration of the ADA inhibitor coformycin into the rat TMN, where ADA is dominantly expressed, increases SWS, further supporting a hypnotic role for adenosine.

### Effects of A_1_ Receptors and Sleep Homeostasis

Adenosine acting through A_1_Rs facilitates sleep as non-selective and selective A_1_R agonists increase sleep and SWA (Radulovacki et al., 1984; Benington et al., 1995), whereas A_1_R antagonists decrease sleep and SWA (Virus et al., 1990; Methippara et al., 2005; Thakkar et al., 2008). Furthermore, A_1_R antagonism within the BF reduces the homeostatic sleep and SWA response following acute sleep deprivation (Gass et al., 2009). Conditional KO of A_1_Rs predominantly affecting forebrain glutamatergic neurons prevents sleep deprivation-induced increases in SWA, indicating that A_1_Rs are necessary for normal sleep homeostasis (Bjorness et al., 2009). In mixed background mice with constitutive KO of A_1_Rs, the normal sleep homeostatic response is maintained as measured by slow wave energy [SWE; SWA (0.5–4.5 Hz) × time] in SWS (Stenberg et al., 2003). Further, acute application of a selective A_1_R antagonist blocks the homeostatic response of increased SWS-SWA in sleep-deprived wild-type mice, but is ineffective in the constitutive KO mice under the same conditions (Stenberg et al., 2003). This finding suggests the presence of compensatory mechanisms in mice with constitutive KO that were not present in mice with conditional KO. Sleep facilitation via A_1_Rs occurs through inhibition of wake-active neurons in several brain areas, including both the brainstem and forebrain regions of the cholinergic arousal system [mesopontine tegmentum (Rainnie et al., 1994) and BF (Alam et al., 1999; Thakkar et al., 2003)], and the lateral hypothalamus containing hypocretin/orexin neurons (Liu and Gao, 2007). Additionally, administration of a selective A_1_R agonist into the TMN decreases histamine in the frontal cortex while increasing sleep and SWA (Oishi et al., 2008), suggesting that adenosine also inhibits activity of this wake-promoting neurotransmitter system. An additional mechanism by which adenosine facilitates sleep through A_1_Rs is by disinhibiting sleep-active neurons in the VLPO and anterior hypothalamic area (Chamberlin et al., 2003; Morairty et al., 2004). Finally, A_1_Rs mediate homeostatic sleep pressure based on astrocytic gliotransmission (Halassa et al., 2009) and as part of a glial-neuronal circuit (Bjorness et al., 2016).

Prolonged waking through sleep deprivation increases the expression of A_1_Rs in both humans and rodents (Basheer et al., 2007; Elmenhorst et al., 2007), with expression levels normalizing after recovery sleep in humans (Elmenhorst et al., 2017).

As mentioned above, SWA power is the primary indicator of homeostatic sleep need. SWA power reflects both the number of cells firing at SWA frequencies, which is an intrinsic feature of thalamocortical neurons (McCormick and Pape, 1990; Dossi et al., 1992), and the synchronicity of firing across neurons, which is a circuit effect involving cortical neurons, thalamocortical neurons, and neurons of the reticular nucleus of the thalamus (Steriade et al., 1993). Activation of A_1_Rs influences SWA by both direct and indirect mechanisms; the direct mechanism is based on presynaptic inhibition of cortical and thalamic neurons, which results in relative functional deafferentation along with an A_1_R-induced increase in whole cell, GIRK channel conductance and decreased hyperpolarization activated currents (Ih), such that adenosine enhances slow oscillations in thalamocortical neurons (Pape, 1992). The indirect mechanism is a reduction of cholinergic tone by A_1_R-mediated inhibition of cholinergic arousal neurons (Rainnie et al., 1994; Porkka-Heiskanen et al., 1997). Acetylcholine inhibits slow oscillation in thalamocortical neurons (Curro Dossi et al., 1991; Steriade et al., 1991; McCormick, 1993); thus reduction of cholinergic tone is permissive for the expression of SWA.

### Effects of A_2A_ Receptors and Control of Arousal

Infusion of the selective A_2A_R agonist CGS21680 into the subarachnoid space below the ventral surface region of the rostral BF in rats or into the lateral ventricle of mice produces robust increases in SWS and REM sleep (Satoh et al., 1996; Urade et al., 2003). *In vivo* microdialysis experiments, infusing CGS21680 into the BF dose-dependently decreases histamine release in the frontal cortex and medial preoptic area, and increases the release of GABA in the TMN, but not in the frontal cortex (Hong et al., 2005). Infusion of the GABA antagonist picrotoxin into the TMN attenuates the CGS21680-induced inhibition of histamine release, suggesting that the A_2A_R agonist induces sleep by inhibiting the histaminergic system through increasing the release of GABA in the TMN. Intracellular recordings of VLPO neurons in rat brain slices demonstrated that two distinct types of VLPO neurons exist in terms of their responses to serotonin and adenosine. VLPO neurons are inhibited by noradrenaline, acetylcholine, and an A_1_R agonist, whereas serotonin inhibits type-1 neurons, but excites type-2 neurons. An A_2A_R agonist post-synaptically excites type-2, but not type-1, neurons. These findings suggest that type-2 neurons are involved in initiating sleep, whereas type-1 neurons may contribute to sleep consolidation, because they are only activated in the absence of inhibitory effects from wake-inducing systems (Gallopin et al., 2005).

Administration of CGS21680 into the rostral BF, however, produces c-fos expression not only in the VLPO, but also within the NAc shell and the medial portion of the olfactory tubercle (Satoh et al., 1999; Scammell et al., 2001). Direct infusion of the A_2A_R agonist into the NAc induces SWS that corresponds to approximately 75% of the sleep amount measured when the A_2A_R agonist is infused into the subarachnoid space (Satoh et al., 1999). This observation may indicate that activating A_2A_Rs within or close to the NAc induces sleep. Acting opposite to adenosine, caffeine, which is the most widely consumed psychostimulant in the world, enhances wakefulness because it acts to antagonize both A_1_R and A_2A_R subtypes. At doses commonly consumed by humans, caffeine partially (estimated as 25–50%) and non-selectively (similar affinity for both A_1_Rs and A_2A_Rs) blocks adenosine receptors (Fredholm et al., 1999). Experiments using mice with global genetic A_1_R and A_2A_R KO revealed that A_2A_Rs, but not A_1_Rs, mediate the wakefulness-inducing effect of caffeine (Huang et al., 2005), while single nucleotide mutations of the A_2A_R gene confer sensitivity to caffeine and sleep deprivation (Bodenmann et al., 2012). The specific role of A_2A_Rs in the striatum was investigated in conditional A_2A_R KO mice based on the Cre/lox technology and local infection with AAV carrying short-hairpin RNA of the A_2A_R to silence the expression of the receptor. Selective deletion of the A_2A_Rs in the NAc shell blocked caffeine-induced wakefulness (Lazarus et al., 2011).

For caffeine to be effective as an A_2A_R antagonist, adenosine must tonically activate excitatory A_2A_Rs within the NAc shell. This activation likely occurs in the NAc shell because A_2A_Rs are abundantly expressed throughout the striatum, including the NAc shell and sufficient levels of adenosine are available under basal conditions (Rosin et al., 1998; Svenningsson et al., 1999). A recent study showed that chemogenetic or optogenetic activation of NAc A_2A_R core neurons projecting to the VP in the BF strongly induces SWS, whereas chemogenetic inhibition of these neurons prevents sleep induction, but does not affect homoeostatic sleep rebound (Oishi et al., 2017b). Interestingly, motivational stimuli suppress sleep and inhibit the activity of VP-projecting NAc A_2A_R neurons. In addition, another recent study revealed that adenosine is a plausible candidate molecule for activating NAc core A_2A_R neurons to induce SWS because elevated adenosine levels in the NAc core promote SWS via A_2A_Rs (Zhou et al., 2019).

The sleep-gating ability of the NAc indirect pathway may explain the tendency toward falling asleep in boring situations. Interestingly, excessive daytime sleepiness is common in children with attention-deficit/hyperactivity disorder, who frequently start napping or daydream when they are bored (Weinberg and Brumback, 1990). Dopamine produced by VTA neurons has a key role in processing reward, aversive, or cognitive signals (Wise, 2004; Bromberg-Martin et al., 2010; Schultz, 2015), and projections from VTA dopaminergic neurons to the NAc, commonly known as the mesolimbic pathway, constitute a well-characterized reward circuit in the brain (Russo and Nestler, 2013; Volkow and Morales, 2015). Two independent studies recently examined the contribution of VTA dopaminergic neurons to wakefulness under baseline conditions by chemogenetic inhibition. One study found that chemogenetic inhibition of VTA dopamine neurons decreases the amount of wakefulness, thus suggesting that these neurons are necessary for baseline wakefulness in mice (Eban-Rothschild et al., 2016). The other study showed that chemogenetic inhibition of VTA dopamine neurons does not significantly affect wakefulness at baseline in mice (Oishi et al., 2017a). A plausible explanation for the differences in the observations in these studies is different ectopic Cre expression (Lammel et al., 2015) in the midbrain of the tyrosine hydroxylase-Cre mice used by Eban-Rothschild et al. (2016) or the dopamine transporter-Cre mice used by Oishi et al. (2017a).

## Unified Model of Sleep-Wake Regulation: Gating of Sleep Homeostasis by Arousal

**FIGURE 2:**
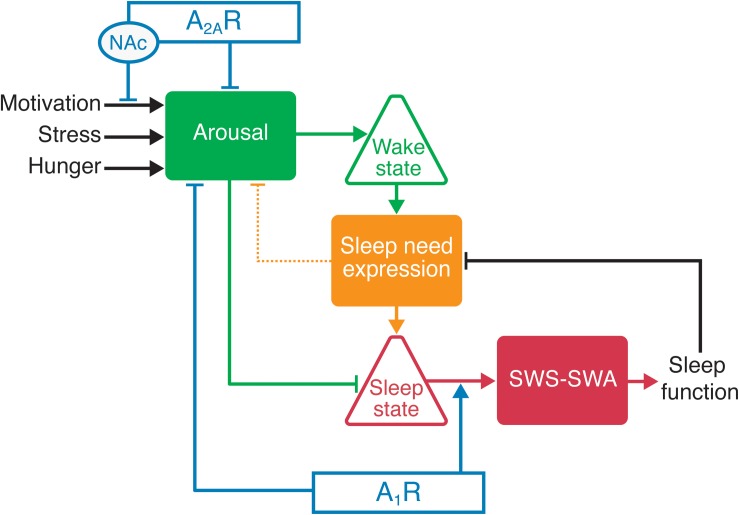
Adenosine receptors influence sleep/wake behavior by modulating the arousal level through A_2A_Rs or A_1_Rs and the sleep need through A_1_Rs. Increased activity of the arousal centers promotes wakefulness. For example, activation of A_2A_Rs in the nucleus accumbens (NAc) and hypothalamus facilitates sleep through the inhibition of arousal-promoting neurons. The duration of wake time positively correlates with sleep need and the buildup of extracellular adenosine. The buildup of adenosine in the cortex and thalamus increases SWS-SWA through the activation of A_1_Rs. Sleep need also increases the probability of a state change from wake to sleep, primarily by decreasing arousal center activity (in part by activating A_1_Rs in arousal centers and A_2A_Rs in the NAc). The sleep state is permissive for sleep function that resolves the sleep need (as sleep function is accomplished), as reflected by the resolution of rebound SWS-SWA.

As knowledge of the molecular and circuit bases of sleep/wake regulation expands, new roles of adenosine receptors in modulating different aspects of sleep emerge. For example, A_2A_Rs appear to promote sleep by suppressing arousal, whereas sleep need and the response to sleep deprivation are mediated by A_1_Rs, and these receptors may thus play a crucial role in the function of sleep. In light of the dissociable effects of adenosine for gating sleep and mediating sleep need at the receptor level, we propose a model of sleep-wake regulation in which the sleep state is regulated by arousal when an organism must consolidate wakefulness in response to environmental changes (Figure 2). A typical example is motivated behavior that efficiently suppresses sleep of all stages and produces arousal by utilizing mesolimbic dopaminergic systems, whereas the wake state is suppressed in the absence of motivating stimuli by activation of A_2A_Rs in the NAc (Oishi and Lazarus, 2017; Oishi et al., 2017b). The circadian and hypothalamic feeding systems have indirect influences by driving internally generated arousal, e.g., increasing motivation to forage according to the circadian phase. Thus in the absence of motivating/external arousing stimuli, the loss of the arousing influence of the circadian system (the sleep phase) may be sufficient to allow transition to sleep. On the other hand, sleep is necessary for SWS-SWA to facilitate the expression of sleep need and for the resolution of sleep need, a process in which A_1_Rs play a crucial role.

## Conclusion

Adenosine is a well-known somnogenic substance that affects normal sleep-wake patterns. While the source of the adenosine involved in sleep remains poorly understood, the metabolism of adenosine in the CNS is significantly mediated by adenosine kinase, which modulates the concentration of adenosine at neuronal A_1_R sites. Similarly, adenosine promotes sleep by several mechanisms in various locations via A_1_Rs or A_2A_Rs.

Adenosine receptor stimulation should be considered as a potential treatment for insomnia. Insomnia is a sleep disorder affecting millions of people around the world and frequently co-occurs with a wide range of psychiatric disorders (Roth, 2007; de Zambotti et al., 2018; Seow et al., 2018). Although A_2A_R agonists strongly induce sleep, classical A_2A_R agonists have adverse cardiovascular effects and cannot be used clinically to treat sleep disorders. Moreover, the development of adenosine analogs for treating central nervous system disorders, including insomnia, is hampered by the poor transport of these drugs across the blood-brain barrier. A small blood brain barrier-permeable monocarboxylate was recently demonstrated to induce sleep by enhancing A_2A_R signaling in the brain, and surprisingly did not exhibit the typical cardiovascular effects of A_2A_R agonists (Korkutata et al., 2017). Therefore, molecules that allosterically enhance A_2A_R signaling could help people with insomnia fall asleep and may also be a potential treatment for psychiatric illness. Similarly, molecules that enhance A_1_R signaling might enhance sleep efficiency.

## Author Contributions

All authors wrote the review, and approved it for publication.

## Conflict of Interest Statement

The authors declare that the research was conducted in the absence of any commercial or financial relationships that could be construed as a potential conflict of interest.
